# Trends in Studies on Transesophageal Echocardiography in Emergency Medicine: A Scoping Review

**DOI:** 10.5811/westjem.24870

**Published:** 2025-05-14

**Authors:** Bor-Yuan Tseng, Chih-Jui Yang, Jen-Tang Sun, Yiju Teresa Liu, Kabir Yadav, Yu-Lin Hsieh, Sheng-En Chu, Chen-Wei Lee, Yi-Kung Lee, Tou-Yuan Tsai

**Affiliations:** * Tzu Chi University, School of Medicine, Hualien, Taiwan; † Far Eastern Memorial Hospital, Department of General Medicine, New Taipei City, Taiwan; ‡ Far Eastern Memorial Hospital, Department of Emergency Medicine, New Taipei City, Taiwan; § Cardinal Tien Junior College of Healthcare and Management, Department of Nursing, Yilan, Taiwan; ¶ Brigham and Women’s Hospital, Department of Medicine, Boston, Massachusetts; || Harbor-UCLA Medical Center, Department of Emergency Medicine, Torrance, California; # Lundquist Institute for Biomedical Innovation, Torrance, California; ** Harvard Medical School, Boston, Massachusetts, USA; †† Institute of Emergency and Critical Care Medicine, College of Medicine, National Yang Ming Chiao Tung University, Taipei, Taiwan; ‡‡ Dalin Tzu Chi Hospital, Buddhist Tzu Chi Medical Foundation, Department of Emergency Medicine, Chiayi, Taiwan; §§ Lunghwa University of Science and Technology, Taoyuan City, Taiwan

## Abstract

**Background:**

Transesophageal echocardiography (TEE) has been introduced in resuscitative scenarios in recent decades, with a growing number of emergency physicians learning, performing, and studying resuscitative TEE.

**Objective:**

Our goal was to characterize publishing trends regarding TEE use in emergency medicine (EM) and to investigate the increasing interest in potential applications of TEE in emergency departments (ED).

**Methods:**

We retrieved published research associated with the use of TEE in EM from the Web of Science database from inception to December 31, 2023. We analyzed trends based on the number of articles published annually. To systematically map trends related to TEE in emergency medicine (EM), we extracted data on the number of unique EM TEE practitioners, institutions performing EM TEE, study topics, and other characteristics from research articles and case reports. To better reflect research trends, we exclusively conducted subgroup analysis on the research articles. We used linear regression analysis to analyze trends and conducted checkpoints on the timelines.

**Results:**

Of the 964 titles and abstracts screened, we included 99 eligible published articles after careful review. Articles related to EM TEE increased from one article in 1991 to 20 articles in 2023, and the rate of publication has increased rapidly since 2018 (+12.4 publications per year, 95% confidence interval [CI] 9.8–15.0, P<0.001). The number of EM TEE practitioners and EM TEE-performing institutions underwent a rapid expansion with an inflection point between 2018–2020, with a rate of +91.7 practitioners per year and +36.5 institutions per year. Subgroup analysis revealed a similar trend in the published research articles. The most common indications for EM TEE were cardiac arrest (72.7%), shock (13.1%), and procedural guidance (11.1%). The United States published the majority of EM TEE-related articles (51.5%).

**Conclusion:**

The present study highlights that TEE-related articles in EM continue to accelerate. Among the indications for TEE, cardiac arrest remains the most frequently discussed. This scoping review provides insights into the expanding interest and applications of TEE in the field of EM.

## INTRODUCTION

Ultrasonography has been applied to the clinical practice of emergency medicine (EM) since 1990.^1^ Emergency physicians (EP) have been trained in the use of point-of-care ultrasonography (POCUS) since 1994, when it was included in the core content of EM residency programs.^2^, ^3^ In recent decades, POCUS has been widely implemented for diagnosis and procedures in EM.^4^ According to the emergency ultrasound guidelines published by the American College of Emergency Physicians (ACEP) in 2023, POCUS has a diverse range of applications, including assessment, investigation and procedural guidance. 5

Using the same goal-directed framework as clinical ultrasound applications, focused or resuscitative transesophageal echocardiography (TEE) has been increasingly used to evaluate critically ill patients in emergency settings. Research on TEE in EM was first published in the 1990s, with articles describing the use of TEE to diagnose aortic and cardiac diseases.^6^ Since 2008, EPs started to use TEE in cardiopulmonary resuscitation, revealing its feasibility, safety, and clinical impact throughout the course of ongoing resuscitative efforts.^7^ An increasing number of EPs are employing TEE as a tool in diagnosis, prognosis, therapeutic guidance, and monitoring for cardiac arrest resuscitation.^8^ Since 2018, guidelines have been published to assist EPs in acquiring the equipment and skills required to successfully incorporate TEE into clinical practice.^5^, ^9^

Although there appears to be increased use of resuscitative TEE in EDs, little attention has been given to the expanded application of TEE in EM. Therefore, in this scoping review we aimed to systemically map the publication trends associated with TEE in EM. Additionally, we sought to describe the characteristics of published articles related to TEE.

## METHODS

### Study Design and Setting

In this scoping review, we investigated all the publication and citation data retrieved from the Web of Science (WoSc) database before December 31, 2023. The study protocol was approved by the Institutional Review Board of Dalin Tzu Chi Hospital, Buddhist Tzu Chi Medical Foundation, Taiwan (No. B11301004). The study adheres to the Preferred Reporting Items for Systematic Reviews and Meta-analyses extension for scoping reviews (PRISMA-SCR) guidelines.^10^ The final protocol was registered prospectively with the Open Science Framework (https://osf.io/4fsnk).

### Article Selection and Assessment

Transesophageal echocardiography is defined as a specific ultrasound technology that places the ultrasound transducer inside the esophagus to acquire exceptionally detailed images of the cardiac and vascular anatomy. 6 Emergency medicine focuses on the initial evaluation and treatment of any patient requiring expeditious medical and surgical care in a hospital-based or freestanding emergency department (ED), urgent care clinic, or prehospital setting (eg, an emergency medical response vehicle or a disaster site).^11^ We searched the literature published from inception through December 31, 2023, in the WoSc database using the following keywords: “transesophageal echocardiography,” “TEE,” and “emergency.” (The search was performed March 1, 2024) We searched for keywords and linked the search terms with logical Boolean operators in titles, abstracts, and keywords according to the type of article.^12^ We also extended our search to include gray literature through manual exploration. To be included in the review, studies had to explicitly demonstrate the performance of TEE in EM settings. We excluded case animal studies, studies not in English, studies in which TEE was not performed on the same day as the ED visit, and those without a clear description of the EM setting.

Two reviewers (BYT and CJY) independently screened the titles and abstracts of all articles that met the inclusion criteria in the search strategy. They subsequently discussed the results and updated a master list of studies. The full texts of potentially eligible studies were retrieved and assessed for eligibility by the same reviewers. Inter-reviewer disagreements were resolved by consensus and, if necessary, a third reviewer (TYT) was consulted. Two reviewers independently abstracted the following data from the included articles: name of the first author; year of publication; number of citations; country of origin; article categories; publishing journal name; article topic; location, type, and number of practitioners; objectives; indication for which TEE was performed; and hospital name(s). We determined country of origin based on the location of the research. Articles were classified by WoSc categories as case reports, research articles, reviews, editorials, or meeting abstracts. The topic of each article was organized into four principal domains: diagnosis; intervention (procedure guidance); education; and safety. Indications for TEE were classified as cardiac arrest, shock, procedural guidance, trauma and cardiac diseases (eg, valvular thrombi), in accordance with prior studies.^6^,^9^

### Outcome Measurement

The primary outcome was the number of TEE-related articles published annually in EM, which we used to evaluate the trend in publication rate during the study period. The number of citations for each article was retrieved from the WoSc database. To characterize global trends in the capacity for EM TEE performance, we also conducted a systematic assessment of the annual changes in the reported numbers of EM TEE practitioners and EM TEE-performing institutions. The number of EM TEE practitioners was defined as the number of practitioners who both performed TEE and were involved in data collection, serving as an indicator of the expansion of EM TEE application worldwide. We abstracted the practitioners’ numbers from original articles by using the following guidelines: 1) based on article description, if details provided; 2) contacting the first and corresponding authors for detailed number by e-mail; and 3) if there was no reply, we estimated based on the number of TEE cases and the number of authors listed in the article. If the number of TEE cases was more than the number of authors, we considered all authors as TEE-performing physicians. If the number of TEE cases was fewer than the number of authors, we first counted the corresponding and first authors as TEE-performing physicians, followed by the second author, and so on, in that order. For authors with multiple published articles, the earliest one published was considered in the event of duplicates. We defined the TEE-performing institution as the hospital or center where TEE was performed. We applied the same calculation method to estimate the number of TEE-facilitating institutes. If more than two published studies originated from the same institution, we extracted only the earliest and removed the duplicate entries. Review articles were excluded from the data of practitioners and institutions. In addition, to reflect the current global research trends regarding the use of TEE in EM, we analyzed research articles focusing on the annual publication trends, the number of EM TEE practitioners, and EM TEE-performing institutions in the subgroup analysis. Other outcomes included the geographic distribution of papers based on country, number of citations of each article, proportion of articles published in dedicated EM journals, type of article, setting, specialty of TEE practitioners, participants, and indications for TEE. Clinical practice characteristics, such as setting, specialty of TEE practitioners, and population for TEE, were extracted only from observational studies and case reports. We conducted subgroup analysis exclusively on the research articles.

### Data Analysis

As this study was a scoping review, we reported only descriptive statistics for the distribution of the number of articles and citations. To clearly present the trends in outcomes, we used the slope (β) of linear regression curves to analyze trends in the number of published studies, EM TEE practitioners, and EM TEE-performing institutions, and we calculated the 95% confidence intervals (CI) of β. Additionally, if the trends of interest did not follow a simple linear pattern, we used the “Rbeast” package to identify any inflection points.^13^ A *P*-value <0.05 was considered statistically significant. We conducted all analyses using R statistical software v 4.2.3 (R Foundation for Statistical Computing, Vienna, Austria).

## RESULTS

### Search Results

An initial search of the WoSc database yielded 964 published articles. We excluded 852 articles based on screening titles and abstracts for irrelevant topics, unavailable data, and duplicate records. The remaining 112 full-text articles were retrieved and assessed for eligibility with 13 potentially relevant studies excluded after full-text review because they had “emergency” in the abstracts but did not perform TEE on the same day of an ED visit or were unrelated to an emergent presentation (Supplementary Figure S1). After careful review, 99 published articles met all the eligibility criteria (Supplementary Table S1). In total, 33 (33.3%) articles were categorized as research articles, 23 (23.3%) as case reports or series, and 18 (18.2%) as review articles (Supplementary Table S2). In the analysis of topics within TEE-related literature, 77.8% (77/99) of articles employed TEE for diagnostic purposes, 15.2% (15/99) focused on TEE training and education, 7.1% (7/99) applied TEE for procedural guidance, and 3.0% (3/99) addressed TEE safety in clinical settings. Overall, the total, average, and median numbers of citations were 1,284, 13.0, and 2, respectively.

### TEE-related Publication Trends, Trends in TEE Practitioners and TEE-performing Institutions

**Figure 1:**
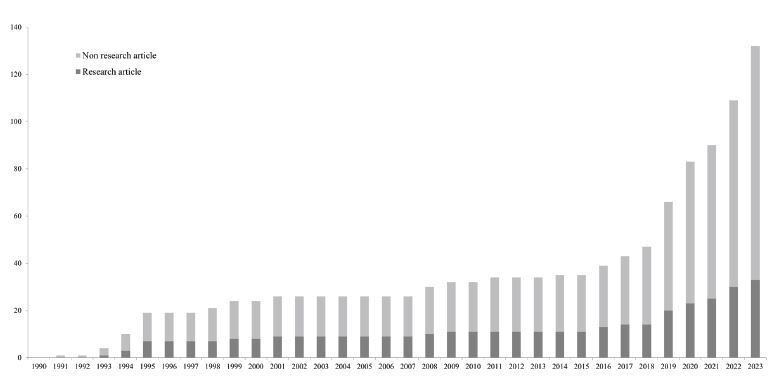
Cumulative number of published articles and research on transesophageal echocardiography in emergency medicine settings from 1990–2023.

Concerning the primary outcome, the number of TEE-related published articles on EM increased from 1 in 1991 to 20 in 2023 (Figure 1). The number of publications has been increasing steadily since 1991 with an increasing rate of publications of 1.8 per year (95% CI 1.3–2.2, *P*<0.001). The inflection point was in 2018, and the number of published articles has increased more rapidly since 2018, with an increasing rate of publication of 12.4 per year (95% CI 9.8–15.0, *P*<0.001). When focusing specifically on research articles, we observed a similar trend and inflection point in the number of articles.

**Figure 2:**
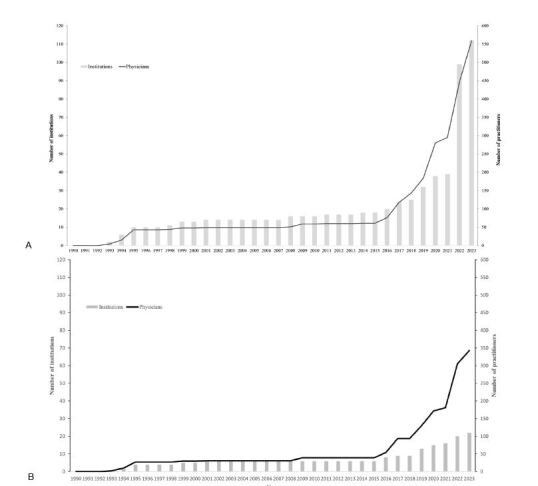
Global trends in transesophageal echocardiography (TEE) clinicians and TEE-facilitated institutions as analyzed in published papers (A), with specific analysis in research articles (B).

We calculated trends in the number of TEE practitioners and institutions from 81 observational studies and case reports. Of these, 70 articles provided details based on descriptions within the paper, two were clarified by contacting the corresponding authors via email, and the remaining nine lacked specific details, prompting estimation based on the number of TEE cases and authors listed in the articles. The first article to calculate the number of TEE practitioners and institutions was published in 1993. The number of TEE practitioners in EM has experienced significant growth, increasing from four EM TEE practitioners described in published articles in 1993 to 559 practitioners in 2023, with a rate of increase of 8.9 practitioners per year (95% CI 5.7–12.0, *P*<0.001). Concurrently, the number of EM TEE-performing institutions in EM also demonstrated a sustained increase from two institutions in 1993 to 112 institutions in 2023, with a rate of increase of 1.6 institutions per year (95% CI 1.0–2.2, *P*<0.001). There is an inflection point between 2018–2020 where both EM TEE practitioners and EM TEE-performing institutions underwent a rapid expansion after this period (Figure 2A), with an increasing rate of 91.7 practitioners per year (after 2018) and 36.5 institutions per year (after 2020). When focusing specifically on research articles, a similar trend and inflection point were observed (Figure 2B). The number of TEE practitioners increased at a rate of 50.8 per year after an inflection point in 2018, while the number of TEE-performing institutions rose at a rate of 2.1 per year after an inflection point in 2016.

### Characteristics of the TEE-related Publications

The 99 retrieved publications originated from 15 countries, and most studies were from the United States (51, 51.5%), followed by South Korea (11, 11.1%), Canada (11, 11.1%), and Taiwan (7, 7.1%) (Table 1). The articles were published in 36 journals, the majority of which were published in the *Annals of Emergency Medicine* (18, 18.2%) and the *American Journal of Emergency Medicine* (15, 15.2%) (Supplementary Table S3).

To present the current status of EM TEE in published articles in more detail, we extracted characteristics of this modality in clinical practice from observational studies and case reports. Overall, 77 of 81 (95.1%) non-review studies were performed in EDs, six (7.4%) in the intensive care unit, and three (3.7%) in prehospital settings (Table 2). The EM TEE was primarily performed by EPs (56, 69.1%), with 18 (22.2%) of the articles mentioning that EM TEE was performed by ED residents. In the subgroup analysis of 33 research articles on TEE in EM, 32 articles (97.1%) reported TEE being performed in EDs, with 30 articles (90.9%) indicating that the procedures were conducted by EPs.

**Figure 3:**
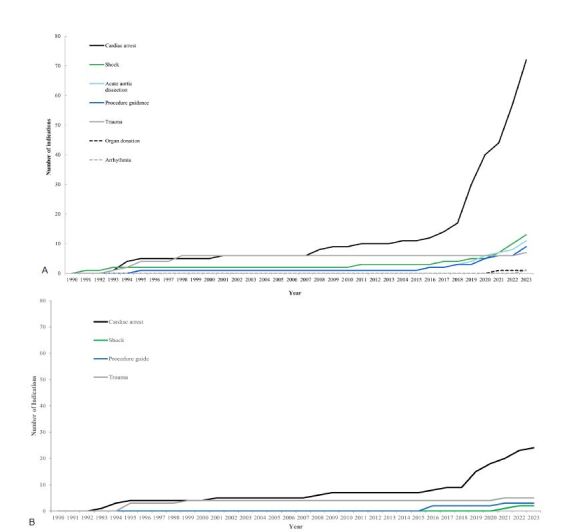
Timeline of indications for transesophageal echocardiography as analyzed in published papers (A), with specific analysis in research papers (B).

The EM TEE was predominantly used for adult patients (56, 69.1%). Five articles reported on the use of TEE in the pediatric population. However, these studies were conducted only on adolescents and not on younger children.^14^–^18^ Owing to the different sizes of EM TEE devices used in pediatric and adult populations, there are currently no articles exploring the application of EM TEE in young children with cardiac arrest.^19^ The most common indications among all studies for TEE in EM were cardiac arrest (72, 72.7%); shock (13, 13.1%); procedural guidance (11, 11.1%); and aortic disorders (10, 10.1%) (Supplementary Table S4). It is worth noting that among the 16 articles published before 2000, 10 specifically focused on the indication of aortic disorders. Since 2008, articles focusing on cardiac arrest as an indication have become predominant, and the number of articles has exponentially increased since 2018 (Figure 3A). A similar trend was observed in research articles (Figure 3B).

## DISCUSSION

To our knowledge, this is the first study to evaluate the trends in TEE-related research of EM and analyze their characteristics. Our study provides a quantitative analysis of the evolving publication trends of TEE in EM. We observed a marked increase in the number of published articles, as well as the number of institutions and TEE practitioners described in these articles, after an inflection point around 2018. Most EM TEE publications were authored by EPs, with a significant focus on indications for cardiac arrest. A similar trend was observed when analyzing research articles specifically.

The first TEE-related article on EM was published in 1991, and EM TEE was primarily used to diagnose aortic dissection and blunt chest injuries in the 1990s. 6, 14, 20, 21 In the late 1990s and early 2000s, there were some TEE-related articles during this period focused on studying physiology of the cardiac pump theory rather than the application of TEE in clinical practice.^22^–^24^ In 2008 Blaivas et al reported a case series of TEE with six cases, offering distinct advantages of TEE over TTE in patients with cardiac arrest and undergoing cardiopulmonary resuscitation (CPR).^7^ Since then, TEE-related articles have gradually increased and focused on TEE use in assessing CPR quality and cardiac arrest etiologies.^25^,^26^ In 2018, ACEP published clinical guidelines for TEE applications in cardiac arrest resuscitation.^9^ Published articles related to TEE exponentially increased thereafter (Figure 1). In the 2020s Teran et al initiated a multicenter TEE registry study^27^; subsequently, the recorded number of TEE practitioners and institutions facilitating TEE exponentially increased since 2020 (Figure 2).

Similar to the trend observed in our study, a recent North American cross-sectional survey revealed that over 80% of EPs expressed interest in performing TEE in EDs where it is not currently used.^28^ However, the initiation and development of EM TEE programs face multiple barriers, including safety concerns, financial constraints, credentialing/privileges issues, and the absence of TEE-trained EPs or champions. The main barrier for EPs to perform TEE was the concern of associated complications, which aligns with our findings in this scoping review. We observed that many TEE-related articles focus on the safety of TEE use in the ED setting.^29^–^33^ Furthermore, one of our included studies suggested that charging higher fees for TEE-guided resuscitation could promote the development of TEE programs, especially if evidence continues to show that TEE improves resuscitation quality.^28^

Another common barrier is the need for resources to credential and privilege users. Our scoping review also found a growing trend in studies on competency maintenance and quality assurance.^34^,^35^ Interestingly, 16.2% of the included articles focused on educational issues, such as TEE hands-on training, teaching programs, and evaluation of performance (Supplementary Table S2). Furthermore, 18 of the included studies enrolled ED residents (Table 2); eight of these 18 articles focused on educational issues, while in the remaining 10, residents performed TEE based on ACEP training and credentialing recommendations.^9^ These trends suggest that TEE education is an important research area with evaluation of residency training and certification systems to further support expansion of TEE in the ED.

Based on the trend analysis, EM TEE-related studies focus primarily on resuscitation, especially in patients with cardiac arrest. Current guidelines recommend chest compressions on the lower sternum, but factors like cardiomegaly, lung disease, and anatomical variations may limit effectiveness; future studies will explore how TEE improves the chest compression position during CPR, diagnosing the etiology of cardiac arrest, and prognostication of survival.^36^ Currently, three-dimensional (3D) TEE is widely used under many critical conditions.^37^,^38^ As the time required for 3D reconstruction decreases and image quality improves, the application of 3D TEE in resuscitation may play a valuable role in the future.^38^ Moreover, the development of artificial intelligence (AI) to capture and process TEE images should improve the speed and accuracy of this POCUS modality, making it more accessible to clinicians across various specialties.^38^,^40^ With the application of 3D and AI techniques, TEE may be more broadly used in resuscitation and could further expand clinical applications to resource-limited settings in the future.^41^

## LIMITATIONS

The main strength of this scoping review is its quantitative review of current trends in TEE-related publications in EM. However, this study had some limitations. First, our study has a risk of publication bias. Studies tend to be accepted for publication if they have significant findings. Conversely, the lack of benefit for certain indications may be under-represented because of rejection of manuscripts that lack positive, significant, or novel results. Second, the number of EM TEE practitioners and EM TEE-performing institutions may have been underestimated, as some practitioners and institutions do not publish their work or conduct clinical research. Nevertheless, the observed trends in these numbers still provide important insight into this burgeoning field. Third, our study classified countries according to the first author’s affiliation, which likely underestimated the extent of international collaborative research and contributions.

Fourth, the impact of studies conducted after 2023 was underestimated. This resulted from the gap between the paper’s publication and the appearance of citations in other journals. Furthermore, studies published in 2022 may also not have sufficient time to accumulate citations. This phenomenon has been observed in previous studies. 42 Finally, there is a possibility of bias in our data due to the potential misclassification of the setting and physicians involved in TEE administration. Although our objective was to categorize these variables accurately, varying healthcare systems, definitions of healthcare facilities, and specialist roles across countries might have influenced these classifications.

## CONCLUSION

This study highlights the ongoing acceleration in the number and variety of TEE-related published research in EM. Among the indications for TEE, cardiac arrest remains the most frequently discussed, indicating a predominant area of interest within TEE-related publications. This scoping review provides insights into the expanding interest and applications of TEE in the field of EM.

## Supplementary Information





## Figures and Tables

**Table 1:** Distribution of transesophageal echocardiography-related papers published by country.

Country	Number of publications	%	Total times cited
United States	51	51.5	762
South Korea	11	11.1	148
Canada	11	11.1	96
Taiwan	7	7.1	69
Italy	4	4.0	36
United Kingdom	3	3.0	45
United Arab Emirates	2	2.0	16
Germany	2	2.0	2
Malaysia	2	2.0	0
France	1	1.0	105
Turkey	1	1.0	2
Belgium	1	1.0	2
Denmark	1	1.0	2
Ireland	1	1.0	0
Austria	1	1.0	0

**Table 2:** Characteristics of transesophageal echocardiography-related articles.

	Number (%) of articles*		Number of articles focusing on research studies#	%
Setting				
Emergency department	77	95.1	32	97.1
ICU**	6	7.4	5	15.2
Prehospital	3	3.7	2	6.1
Operating room	3	3.7	2	6.1
NA †	3	3.7	0	0
Population				
Adult	56	69.1	25	78.8
Pediatric	5	6.2	3	9.1
NA †	24	29.6	8	24.2
Specialty of Practitioners				
Emergency physician §	56	69.1	30	90.9
Cardiologist	10	12.3	4	12.1
Intensivist	8	9.9	6	15.2
Echocardiographer‡	1	1.0	1	3.0
EMS physician	1	1.0	0	0
NA †	17	21.0	1	3.0

*ICU*, intensive care unit*; NA*, not applicable.

* The number and percentage of published papers were calculated from 81 observational studies and case reports, with some studies reporting multiple characteristics.

# The number and percentage of published papers were calculated from 33 research papers, with some studies reporting multiple characteristics.

** The articles that mentioned the ICU setting also included patients who underwent TEE in the ED within the same study.

† Some data were not applicable because the study was either a simulation or an educational study.

§ Eighteen of the 56 studies demonstrated that emergency medicine residents and fellows provided TEE, contributing to 22.2% of the extracted data in TEE-related studies.

‡ This study (Cohn, SM, 1995)^21^ did not mention the specialty of the echocardiographers.
