# Supplementary figures and images for: Trends in Studies on Transesophageal Echocardiography in Emergency Medicine: A Scoping Review
Source: West J Emerg Med. 2025 May 14;26(3):469–77. doi: 10.5811/westjem.24870 (PMC12208095; doi:10.5811/westjem.24870)

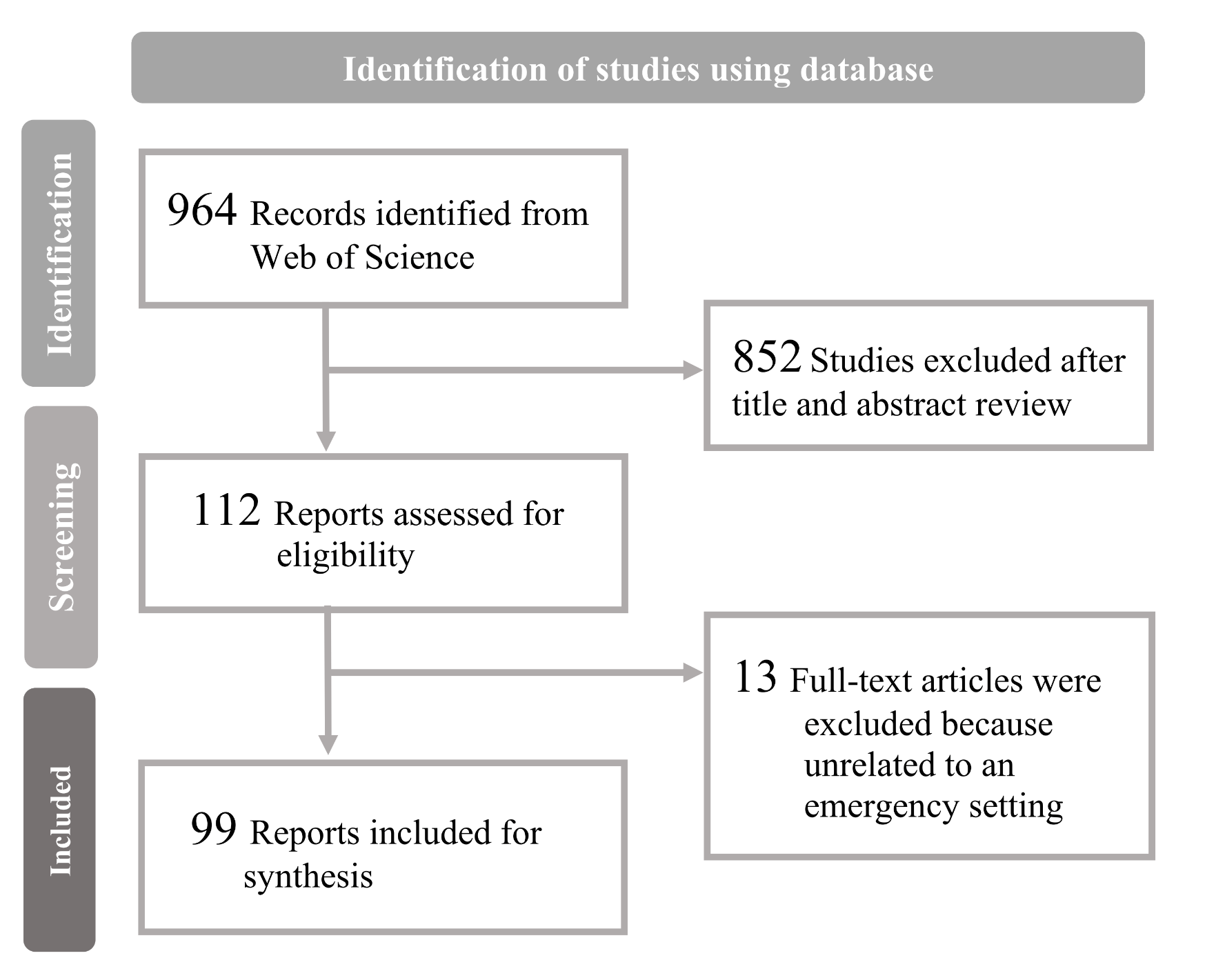

Supplement: Supplementary file 1 [file wjem-26-469-s001.docx]
