# Supplementary material for: Trends in Studies on Transesophageal Echocardiography in Emergency Medicine: A Scoping Review
Source: West J Emerg Med. 2025 May 14;26(3):469–77. doi: 10.5811/westjem.24870 (PMC12208095; doi:10.5811/westjem.24870)
Supplement: Supplementary file 2 [file wjem-26-469-s002.docx]

**Supplemental Online Content**

**Supplementary Table S1.** The transesophageal echocardiography related publications in emergency medicine, ordered by citation numbers**2**

**Supplementary Table S2.** Transesophageal echocardiography-related publications by article type **11**

**Supplementary Table S3.** Journals that published transesophageal echocardiography-related publications in emergency medicine by number of publications. **12**

**Supplementary Table S4.** Indications for transesophageal echocardiography according to the included publications **14**

**Supplementary Table S1.** The transesophageal echocardiography related publications in emergency medicine, ordered by citation numbers

| **No.** | **Title** | **First**  **author** | **Country** | **Journal name** | **Year** | **Overall cited times** |
| --- | --- | --- | --- | --- | --- | --- |
|  | Transesophageal Echocardiography in the Diagnosis of Traumatic Rupture of the Aorta | Smith, M.D. | United States | New England Journal of Medicine | 1995 | 191 |
|  | Role of Transesophageal Echocardiography in the Diagnosis and Management of Traumatic Aortic Disruption | Vignon, P. | France | Circulation | 1995 | 105 |
|  | Focused Transesophageal Echocardiography by Emergency Physicians is Feasible and Clinically Influential: Observational Results from a Novel Ultrasound Program | Arntfield, R. | Canada | The Journal of Emergency Medicine | 2016 | 79 |
|  | Transesophageal echocardiography during cardiopulmonary arrest in the emergency department | Blaivas, M. | United States | Resuscitation | 2008 | 78 |
|  | Compression of the Left Ventricular Outflow Tract During Cardiopulmonary Resuscitation | Hwang, S.O. | South Korea | Academic Emergency Medicine | 2009 | 68 |
|  | Physiology of Blood Flow During Cardiopulmonary Resuscitation. A Transesophageal Echocardiographic Study. | Redberg, R.F. | United States | Circulation | 1993 | 64 |
|  | Evaluation of out-of-hospital cardiac arrest using transesophageal echocardiography in the emergency department | Teran, F. | United States | Resuscitation | 2019 | 60 |
|  | Transesophageal Echocardiographic Assessment of Mitral Valve Position and Pulmonary Venous Flow During Cardiopulmonary Resuscitation in Humans | Ma, H.M. | Taiwan | Circulation | 1995 | 45 |
|  | Transesophageal Echocardiography: Guidelines for Point-of-Care Applications in Cardiac Arrest Resuscitation | Fair, J. | United States | Annals of Emergency Medicine | 2018 | 42 |
|  | Transesophageal Echocardiography During Cardiopulmonary Resuscitation Is Associated with Shorter Compression Pauses Compared with Transthoracic Echocardiography | Fair, J. | United States | Annals of Emergency Medicine | 2019 | 34 |
|  | Echocardiography in cardiac arrest: An emergency medicine review | Long, B. | United States | American Journal of Emergency Medicine | 2018 | 32 |
|  | Emergency physician-performed transesophageal echocardiography for extracorporeal life support vascular cannula placement | Fair, J. | United States | American Journal of Emergency Medicine | 2016 | 31 |
|  | Association between left ventricular outflow tract opening and successful resuscitation after cardiac arrest | Catena, E. | Italy | Resuscitation | 2019 | 29 |
|  | The Use of Transesophageal Echocardiography During Cardiac Arrest Resuscitation: A Literature Review | Parker, B.K. | United States | Journal of Ultrasound in Medicine | 2019 | 25 |
|  | Direction of blood flow from the left ventricle during cardiopulmonary resuscitation in humans-its implications for mechanism of blood flow | Kim, H. | South Korea | American Heart Journal | 2008 | 24 |
|  | Changes of aortic dimensions as evidence of cardiac pump mechanism during cardiopulmonary resuscitation in humans | Hwang, S.O. | South Korea | Resuscitation | 2001 | 24 |
|  | Mechanism of closed chest cardiopulmonary resuscitation investigated by transoesophageal echocardiography | Pell, A.C. | United Kingdom | Journal of Accident and Emergency Medicine | 1994 | 24 |
|  | Assessment of the active compression-decompression device (ACD) in cardiopulmonary resuscitation using transoesophageal echocardiography | Pell, A.C. | United Kingdom | Resuscitation | 1994 | 21 |
|  | Complications of transesophageal echocardiography in the ED | Gendreau, M.A. | United States | American Journal of Emergency Medicine | 1999 | 21 |
|  | Exclusion of Aortic Tear in the Unstable Trauma Patient. The Utility of Transesophageal Echocardiography | Cohn, S.M. | United States | Journal of Trauma-Injury Infection and Critical Care | 1995 | 18 |
|  | Critical Care Transesophageal Echocardiography in Patients during the COVID-19 Pandemic | Teran, F. | United States | Journal of the American Society of Echocardiography | 2020 | 18 |
|  | Focused Transesophageal Echocardiography During Cardiac Arrest Resuscitation JACC Review Topic of the Week | Teran, F. | United States | Journal of the American College of Cardiology | 2020 | 18 |
|  | Diagnosis of Coronary Artery Dissection following Blunt Chest Trauma by Transesophageal Echocardiography | Cherng, W.J. | Taiwan | Journal of Trauma-Injury Infection and Critical Care | 1995 | 17 |
|  | Resuscitative Cardiopulmonary Ultrasound and Transesophageal Echocardiography in the Emergency Department | Teran, F. | United States | Emergency Medicine Clinics of North America | 2019 | 17 |
|  | Severe right ventricular contusion mimicking cardiac tamponade: The value of transesophageal echocardiography in blunt chest trauma | Goldberg, S.P. | United States | Annals of Emergency Medicine | 1993 | 16 |
|  | Intra-arrest transoesophageal echocardiographic findings and resuscitation outcomes | Jung, W.J. | South Korea | Resuscitation | 2020 | 14 |
|  | Emergency Physician-performed Transesophageal Echocardiography in Simulated Cardiac Arrest | Byars, D.V. | United States | Western Journal of Emergency Medicine | 2017 | 14 |
|  | Echocardiography in thoracic trauma | Chan, D. | United States | Emergency Medicine Clinics of North America | 1998 | 12 |
|  | Use of transesophageal echocardiography in the detection and consequences of an intracardiac bullet | Limandri, G. | United States | American Journal of Emergency Medicine | 1994 | 11 |
|  | RESCUE transesophageal echocardiography for monitoring of mechanical chest compressions and guidance for extracorporeal cardiopulmonary resuscitation cannulation in refractory cardiac arrest | Giorgetti, R. | United States | Journal of Clinical Ultrasound | 2020 | 9 |
|  | Transoesophageal echocardiography in cardiac arrest: A systematic review | Hussein, L. | United Arab Emirates | Resuscitation | 2021 | 8 |
|  | Bedside ultrasound in cardiac standstill: a clinical review | Hussein, L. | United Arab Emirates | Ultrasound Journal | 2019 | 8 |
|  | Feasibility, utility, and safety of fully incorporating transesophageal echocardiography into emergency medicine practice | Reardon, R.F. | United States | Academic Emergency Medicine | 2022 | 8 |
|  | Safety of Transesophageal Echocardiography Performed by Intensivists and Emergency Physicians in Critically Ill Patients With Coagulopathy and Thrombocytopenia: A Single-Center Experience | Wray, T.C. | United States | Journal of Intensive Care Medicine | 2021 | 8 |
|  | Transesophageal echocardiography identification of aortic dissection during cardiac arrest and cessation of ECMO initiation | Kelly, C. | United States | American Journal of Emergency Medicine | 2019 | 7 |
|  | Temporary transvenous pacer placement under transesophageal echocardiogram guidance in the Emergency Department | Lerner, R.P. | United States | American Journal of Emergency Medicine | 2020 | 7 |
|  | Diagnosis of aortic dissection by transesophageal echocardiography during cardiopulmonary resuscitation | Kim, Y.W. | South Korea | American Journal of Emergency Medicine | 2021 | 6 |
|  | Simulator-based training for learning resuscitative transesophageal echocardiography | Chenkin, J. | Canada | Canadian Journal of Emergency Medicine | 2019 | 6 |
|  | Transesophageal Echocardiography Performed by Intensivist and Emergency Physicians-A 5-Year, Single-Center Experience | Wray, T.C. | United States | Journal of Intensive Care Medicine | 2022 | 6 |
|  | An elderly woman that presents with absent vital signs | Arntfield, R.T. | Canada | Chest | 2014 | 5 |
|  | Acute aortic dissection developed after cardiopulmonary resuscitation: transesophageal echocardiographic observations and proposed mechanism of injury | Lee, D.K. | South Korea | Acute and Critical Care | 2019 | 5 |
|  | Transesophageal Echocardiography at the Golden Hour: Identification of Blunt Traumatic Aortic Injuries in the Emergency Department | Osman, A. | United States | Journal of Emergency Medicine | 2020 | 5 |
|  | Core Ultrasound in REsuscitation (CURE): A novel protocol for ultrasound-assistant life support via application of both transesophageal and transthoracic ultrasound | Chu, S.E. | Taiwan | Resuscitation | 2022 | 4 |
|  | Kinetic analysis of cardiac compressions during cardiopulmonary resuscitation | Kim, Y.W. | South Korea | Journal of Critical Care | 2019 | 4 |
|  | Transesophageal echocardiography (TEE) in cardiac arrest: results of a hands-on training for a simplified TEE protocol | Nazerian, P. | Italy | Ultrasound Journal | 2020 | 4 |
|  | Aortic valve disruption and regurgitation complicating CPR detected by transesophageal echocardiography | Ma, H.M. | Taiwan | American Journal of Emergency Medicine | 1994 | 3 |
|  | Transesophageal echocardiography in emergency medicine and critical care | Shenoy, M.M. | United States | American Journal of Emergency Medicine | 1991 | 3 |
|  | Bedside Identification of Massive Pulmonary Embolism with Point-of-Care Transesophageal Echocardiography | Jelic, T. | Canada | Journal of Emergency Medicine | 2017 | 3 |
|  | The future of cardiopulmonary resuscitation: What if a TEE probe could shock, sense and pace? | Sloth, E. | Denmark | Resuscitation | 2011 | 2 |
|  | Thrombolysis associated with LUCAS (Lund University Cardiopulmonary Assist System) as treatment of valve thrombosis resulting in cardiac arrest | Gottignies, P. | Belgium | American Journal of Emergency Medicine | 2011 | 2 |
|  | Cardiopulmonary resuscitation may cause paradoxical embolism | Kim, S.J. | South Korea | American Journal of Emergency Medicine | 2020 | 2 |
|  | Successful lysis in massive pulmonary embolism with thrombus entrapped in PFO | Kucukseymen, S. | Turkey | American Journal of Emergency Medicine | 2017 | 2 |
|  | Ruptured aorta after blunt chest trauma: rapid diagnosis by transoesophageal echocardiography when radiographic and computed tomographic findings inconclusive | Bruch, C. | Germany | Deutsche Medizinische Wochenschrift | 1998 | 2 |
|  | Kinetics of manual and automated mechanical chest compressions | Colombo, R. | Italy | Resuscitation | 2019 | 2 |
|  | Transesophageal Echocardiography Use During Cardiopulmonary Resuscitation | Shiber, J. | United States | Annals of Emergency Medicine | 2019 | 2 |
|  | TEE guided REBOA deflation following ROSC for non-traumatic cardiac arrest | Kelly, C. | United States | American Journal of Emergency Medicine | 2023 | 1 |
|  | Right-to-Left Shunts Occur During Cardiopulmonary Resuscitation: Echocardiographic Observations | Jung, W.J. | South Korea | Critical Care Medicine | 2022 | 1 |
|  | Echocardiography in cardiac arrest: strengths and limitations | Lazzeri, C. | Italy | European Journal of Emergency Medicine | 2022 | 1 |
|  | A Case for the Use of Transesophageal Echocardiography in the ED Treatment of Cardiac Arrest | Rublee, C. | United States | Chest | 2020 | 1 |
|  | Duration of Pulse Checks Using Point-of-Care Transthoracic Echocardiography versus Point-of-Care Transesophageal Echocardiography versus Palpation | Wagner, B.P. | United States | Annals of Emergency Medicine | 2020 | 1 |
|  | Indications, Clinical Impact, and Complications of Critical Care Transesophageal Echocardiography: A Scoping Review | Prager, R. | Canada | Journal of Intensive Care Medicine | 2023 | 1 |
|  | Transesophageal Echocardiography in Patients in Cardiac Arrest: The Heart and Beyond | Teran, F. | Canada | Canadian Journal of Cardiology | 2023 | 1 |
|  | Resuscitative transesophageal echocardiography in the diagnosis of post-CABG loculated pericardial clot causing cardiac tamponade | Adi, O. | United States | Ultrasound Journal | 2021 | 1 |
|  | Transesophageal echocardiography in the emergency department: A comprehensive guide for acquisition, implementation, and quality assurance | McGuire, D. | United States | Journal of the American College of Emergency Physicians Open | 2022 | 1 |
|  | Critical Care Transesophageal Echocardiography for the Resuscitation of Shock: An Important Diagnostic Skill for the Modern Intensivist | Prager, R. | United Kingdom | Chest | 2023 | 0 |
|  | Managing Cardiac Arrest Using Ultrasound | Gottlieb, M. | United States | Annals of Emergency Medicine | 2023 | 0 |
|  | Response to Echocardiography in cardiac arrest: strengths and limitations | Lien, W.C. | Taiwan | European Journal of Emergency Medicine | 2022 | 0 |
|  | Multicenter Prospective Evaluation of Out-of-Hospital Cardiac Arrest Patients Using Transesophageal Echocardiography: A Preliminary Analysis from The Resuscitative TEE Collaborative Registry | Teran, F. | United States | Annals of Emergency Medicine | 2022 | 0 |
|  | Resuscitative TEE Collaborative Registry: Development and Implementation of a Multicenter Registry for Focused Transesophageal Echocardiography (TEE) in the Emergency Department and Intensive Care Settings | Teran, F. | United States | Annals of Emergency Medicine | 2022 | 0 |
|  | A Brief Cadaver Training Session Is Feasible for Training Novice Users on Transesophageal Echocardiography Transducer Insertion | Horton, R. | United States | Annals of Emergency Medicine | 2018 | 0 |
|  | Failure of serial electrocardiograms to exclude a large anterior myocardial infarction and the role of transesophageal echocardiogram in acute myocardial infarction | Wang, Y.L. | United States | Echocardiography: A Journal of Cardiovascular Ultrasound and Allied Techniques | 1999 | 0 |
|  | A brief history of point-of-care transesophageal echocardiography in the USA | West, F.M. | United States | European Journal of Emergency Medicine | 2021 | 0 |
|  | Proposal for an Amendment to the ACEP Transesophageal Echocardiography Guidelines: The Aortic View | Kelly, C. | United States | Annals of Emergency Medicine | 2023 | 0 |
|  | Transesophageal Echocardiography May Improve Cerebral Perfusion Compared to Transthoracic Echocardiography in Out of Hospital Cardiac Arrest | Chinn, E. | United States | Annals of Emergency Medicine | 2020 | 0 |
|  | Diagnosis of aortic dissection during cardiopulmonary resuscitation. Transthoracic versus transesophageal echocardiography | Hwang, S.O. | South Korea | American Journal of Emergency Medicine | 2020 | 0 |
|  | Evaluation of Performance of Transesophageal Echocardiography by Emergency Medicine Residents After a Single Simulation-Based Training Session | Diaz, O. | United States | Annals of Emergency Medicine | 2022 | 0 |
|  | The Impact of Resuscitative Transesophageal Echocardiography Performed by Emergency Physicians on Diagnosis and Management of Critically Ill Patients | Kegel, F. | Canada | Annals of Emergency Medicine | 2022 | 0 |
|  | The utility of multiple imaging modalities to diagnose acute aortic dissection | Hurley, K.F. | Canada | Canadian Journal of Emergency Medicine | 2008 | 0 |
|  | Putting the Guidelines Ahead of the Evidence for the Use of Transesophageal Echocardiography by Emergency Physicians | Ehrman, R.R. | United States | Annals of Emergency Medicine | 2018 | 0 |
|  | Emergency Physician Learning Curve on Transesophageal Echocardiography Simulator | Lin, J. | United States | Annals of Emergency Medicine | 2020 | 0 |
|  | Optimizing simulator-based training for emergency transesophageal echocardiography: A randomized controlled trial | Chenkin, J. | Canada | Academic Emergency Medicine Education and Training | 2023 | 0 |
|  | A cadaveric model for transesophageal echocardiography transducer placement training: A pilot study | Horton, R.W. | United States | World Journal of Emergency Medicine | 2022 | 0 |
|  | Rescue Transesophageal Echocardiography: A Narrative Review of Current Knowledge and Practice | Efrimescu, C.I. | Ireland | Journal of Cardiothoracic and Vascular Anesthesia | 2023 | 0 |
|  | An Innovative Curriculum For Teaching Transesophageal Echocardiography (TEE) to Emergency Medicine Residents | Field, S. | United States | Journal of Emergency Medicine | 2022 | 0 |
|  | Transesophageal echocardiography in emergency and intensive care medicine Indication and implementation | Stobe, S. | Germany | Medizinische Klinik – Intensivmedizin und Notfallmedizin | 2019 | 0 |
|  | Intra-arrest transesophageal echocardiography during cardiopulmonary resuscitation | Hwang, S.O. | South Korea | Clinical and Experimental Emergency Medicine | 2022 | 0 |
|  | Resuscitative transesophageal echocardiography in the emergency department: a single-centre case series | Kegel, F. | Canada | Scandinavian Journal of Trauma, Resuscitation and Emergency Medicine | 2023 | 0 |
|  | Transesophageal Echocardiography Training of Emergency Physicians Through an E-Learning System | Salerno, A. | United States | Journal of Emergency Medicine | 2020 | 0 |
|  | Transesophageal echocardiography (TEE)-guided transvenous pacing (TVP) in emergency department | Adi, O | Malaysia | Ultrasound Journal | 2023 | 0 |
|  | Hypotensive patient with superior vena cava obstruction diagnosed using resuscitative transesophageal echocardiography | Adi, O | Malaysia | American Journal of Emergency Medicine | 2023 | 0 |
|  | Resuscitative transesophageal echocardiography during the acute resuscitation of trauma: A retrospective observational study | Prager, R | Canada | Journal of Critical Care | 2023 | 0 |
|  | The Learning Curve of Resuscitative Transesophageal Echocardiography Performed by Emergency Physicians for Patients with Out-of-Hospital Cardiac Arrest | Lee, PY | Taiwan | Annals of Emergency Medicine | 2023 | 0 |
|  | Proficiency-Based Simulation Training: Will This Work for Resuscitative Transesophageal Echocardiography? | Huang, W | United States | Annals of Emergency Medicine | 2023 | 0 |
|  | Feasibility of Transesophageal Echocardiography in Out-of-Hospital Cardiac Arrest: A Single Center Pilot Study | Bianconi, K | United States | Annals of Emergency Medicine | 2023 | 0 |
|  | Precise identification of area of maximal compression using transesophageal echocardiography during cardiopulmonary resuscitation | Chang, CJ | Taiwan | Resuscitation | 2023 | 0 |
|  | Hemodynamic impact of chest compression location during cardiopulmonary resuscitation guided by transesophageal echocardiography | Teran, F | United States | Critical Care Medicine | 2023 | 0 |
|  | Seeing the heart of the problem: transesophageal echocardiography in cardiac arrest: a practical review | Wray, TC | United States | International Anesthesiology Clinics | 2023 | 0 |
|  | Focused Ultrasonography in Cardiac Arrest | Kongkatong, M | United States | Emergency Medicine Clinics of North America | 2023 | 0 |
|  | Feasibility of resuscitative transesophageal echocardiography at out-of-hospital emergency scenes of cardiac arrest | Mario Krammel | Austria | Scientific Reports | 2023 | 0 |

**Supplementary Table S2.** Transesophageal echocardiography-related publications by article type

|  | **Number of publications^*^** | **%** |
| --- | --- | --- |
| **Article type** |  |  |
| Research article | 33 | 33.3 |
| Case report or case series | 23 | 23.3 |
| Review | 18 | 18.2 |
| Letter | 10 | 10.1 |
| Meeting abstract | 10 | 10.1 |
| Editorial material | 5 | 5.0 |
| **Study topic** |  |  |
| Diagnosis | 76 | 76.8 |
| Education | 16 | 16.2 |
| Procedure guidance | 7 | 7.1 |
| Safety | 3 | 3.0 |

^*^ Some articles mentioned more than one characteristic.

**Supplementary Table S3.** Journals that published transesophageal echocardiography-related publications in emergency medicine by number of publications.

| **Journal name** | **Number of publications** | **%** | **Total times cited** |
| --- | --- | --- | --- |
| *Annals of Emergency Medicine* | 18 | 18.2 | 107 |
| *American Journal of Emergency Medicine* | 15 | 15.2 | 128 |
| *Resuscitation* | 11 | 11.1 | 242 |
| *Journal of Emergency Medicine* | 5 | 5.1 | 87 |
| *Ultrasound Journal* | 4 | 4.0 | 13 |
| *Circulation* | 3 | 3.0 | 214 |
| *Emergency Medicine Clinics of North America* | 3 | 3.0 | 29 |
| *Journal of Intensive Care Medicine* | 3 | 3.0 | 15 |
| *Chest* | 3 | 3.0 | 6 |
| *European Journal of Emergency Medicine* | 3 | 3.0 | 1 |
| *Academic Emergency Medicine* | 2 | 2.0 | 76 |
| *Journal of Trauma-Injury Infection and Critical Care* | 2 | 2.0 | 35 |
| *Canadian Journal of Emergency Medicine* | 2 | 2.0 | 6 |
| *Journal of Critical Care* | 2 | 2.0 | 4 |
| *Critical Care Medicine* | 2 | 2.0 | 1 |
| *New England Journal of Medicine* | 1 | 1.0 | 191 |
| *Journal of Ultrasound in Medicine* | 1 | 1.0 | 25 |
| *Journal of Accident & Emergency Medicine* | 1 | 1.0 | 24 |
| *American Heart Journal* | 1 | 1.0 | 24 |
| *Journal of The American College of Cardiology* | 1 | 1.0 | 18 |
| *Journal of The American Society of Echocardiography* | 1 | 1.0 | 18 |
| *Western Journal of Emergency Medicine* | 1 | 1.0 | 14 |
| *Journal of Clinical Ultrasound* | 1 | 1.0 | 9 |
| *Acute And Critical Care* | 1 | 1.0 | 5 |
| *Deutsche Medizinische Wochenschrift* | 1 | 1.0 | 2 |
| *Canadian Journal of Cardiology* | 1 | 1.0 | 1 |
| *Journal of The American College of Emergency Physicians Open* | 1 | 1.0 | 1 |
| *Echocardiography: A Journal of Cardiovascular Ultrasound and Allied Techniques* | 1 | 1.0 | 0 |
| *AEM Education and Training* | 1 | 1.0 | 0 |
| *Clinical And Experimental Emergency Medicine* | 1 | 1.0 | 0 |
| *Journal of Cardiothoracic and Vascular Anesthesia* | 1 | 1.0 | 0 |
| *Medizinische Klinik-Intensivmedizin Und Notfallmedizin* | 1 | 1.0 | 0 |
| *Scandinavian Journal of Trauma Resuscitation & Emergency Medicine* | 1 | 1.0 | 0 |
| *World Journal of Emergency Medicine* | 1 | 1.0 | 0 |
| *International Anesthesiology Clinics* | 1 | 1.0 | 0 |
| *Scientific Reports* | 1 | 1.0 | 0 |

**Supplementary Table S4.** Indications for transesophageal echocardiography according to the included publications

|  | **Number of publications^*^** | **%** |
| --- | --- | --- |
| **Indication** |  |  |
| Cardiac arrest | 72 | 72.7 |
| Shock | 13 | 13.1 |
| Acute aortic dissection or other aortic disease | 10 | 10.1 |
| Procedural guidance | 11 | 11.1 |
| Trauma^†^ | 7 | 7.1 |
| Organ donation | 1 | 1.0 |
| Arrhythmia | 1 | 1.0 |
| NA | 3 | 3.0 |

NA, not available.

^*^ Some studies mentioned more than one characteristic.

^†^ Trauma indicates the studies on blunt chest injury or gunshot wound to the chest.
